# Toloese Generates Nitric Oxide through Natural Radiation of Far Infrared Rays, Reducing Serum Glucose, Cholesterol, and Triglycerides

**DOI:** 10.3390/healthcare12121227

**Published:** 2024-06-20

**Authors:** Min-Ho Yeo, Young-Hyeon Lee, Mi-Jin Ryu, Yong-Hak Choi, Hye-Sook Kim, Kyung-Soo Chang

**Affiliations:** 1 Department of Clinical Laboratory Science, Catholic University of Pusan, Busan 46252, Republic of Korea; dualsgh136@naver.com (M.-H.Y.); pw020103@naver.com (M.-J.R.); 2 SayM Co., Ltd., Seongnam-si 13477, Republic of Korea; 3 Division of International Infectious Diseases Control, Faculty of Pharmaceutical Sciences, Okayama University, Tsushima-Naka, Kita-ku, Okayama 700-8530, Japan; hskim@cc.okayama-u.ac.jp

**Keywords:** Toloese, far infrared ray, nitric oxide

## Abstract

Toloese, a bed composition, is formulated with a combination of minerals of various wavelengths by utilizing a specific ratio and particle size. A maturation mixing technique is used without additional compression processes, resulting in the natural formation of numerous fine pores in the bed structure. At 40 °C, far infrared radiation in the range of 5–20 μm is emitted with a 0.916 radiant ratio, and the measured emitted radiant energy is 3.69 × 10^2^ W/m^2^·μm. This study aimed to investigate the influence of far infrared radiation emitted from a Toloese bed on endogenous nitric oxide production. Clinical trials were conducted with 20 healthy adults aged 20 years. Blood samples were collected before and after Toloese bed usage for 1 h daily for 3 weeks. Nitric oxide levels in the saliva and blood of men and women significant increased after they used the Toloese bed for 1 h. Additionally, sweating sharply increased in the upper and lower body regions after Toloese bed usage. No hematological changes or adverse effects were observed, but blood glucose, cholesterol, and triglycerides decreased after Toloese bed usage compared with those before Toloese bed usage. These findings demonstrated that far infrared radiation emitted by the Toloese bed induced endogenous nitric oxide production and contributed to significant reductions in blood glucose, cholesterol, and triglyceride levels.

## 1. Introduction

Nitric oxide (NO) is commonly known as a diatomic free radical generated in automobile exhaust emissions; since its endogenous synthesis in mammalian cells was discovered in 1968, it has been established as a key cellular signaling molecule [1,2,3]. It began to gain attention as a cardiovascular signaling molecule in the medical field in the late 20th century. Initially recognized as a factor that relaxes arterial muscles and regulates blood flow and pressure, it exhibits wide-ranging physiological effects [4,5,6,7,8].

NO is essential for maintaining endothelial cell homeostasis; it is synthesized from L-arginine by nitric oxide synthase (NOS) in endothelial cells [9]. The generated NO regulates vascular contraction and relaxation, inhibits thrombosis, and controls endothelial cell proliferation; consequently, it maintains vascular function stability [10,11]. NOS function declines rapidly starting from the age of 40 years; as a result, endogenous NO production decreases by more than 50% [12,13,14]. This reduction in NO production is associated with a decline in all body functions related to its role; it is also implicated in the development of vascular diseases, such as hypertension, diabetes, and hyperlipidemia [14,15,16].

The Toloese bed used in this study was manufactured by combining minerals such as yellow soil, loess, and tourmaline in specific particle sizes and proportions. It was naturally dried and matured to create numerous fine pores in the bed structure without additional compression processes. Thus, it maintains its structural integrity for an extended period. Although beds made from natural materials such as natural marble or yellow soil have been developed, they emit large amounts of far infrared radiation (FIR) when heated by an electric heater (50–60 °C), which can emit harmful electromagnetic waves; consequently, blood circulation and metabolism are impaired [17]. However, the Toloese, made from a mixture of minerals, emits FIR in the range of 5–20 μm at temperatures similar to human body temperature (37–40 °C). Therefore, it provides the advantage of usability regardless of season.

NO production is promoted by exercise or specific food consumption. This study aimed to investigate the effects of naturally emitted FIR from Toloese beds on endogenous NO levels in healthy adults. The participants were instructed to use Toloese beds for 3 weeks, and changes in endogenous NO levels before and after bed use were examined. Additionally, the effects of increased NO levels on blood pressure, blood glucose, and other hematological and serum parameters before and after Toloese bed usage were compared and analyzed.

## 2. Materials and Methods

### 2.1. Structure of the Toloese Bed

**Figure 1:**
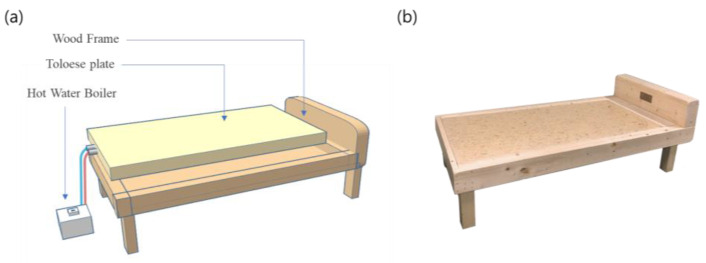
Structure of the Toloese bed. (**a**) Schematic diagram, and (**b**) actual photo of the Toloese bed. The size of the Toloese plate is 840 mm × 1750 mm (width × length).

The Toloese bed used in this study was provided by SayM Co., Ltd. (Seongnam-si, Republic of Korea). The structure and components of the bed are shown in Figure 1. The Toloese plate composition consists of a mixture of minerals, including yellow soil powder (20–40 parts by weight), silica powder (20–40 parts by weight), tourmaline powder (0.5–2 parts by weight), and CMC powder (2–6 parts by weight) as a binder. The Toloese plate is maintained at 37 °C using a hot water boiler. At 37 °C, the emissivity is 0.921 and the radiative energy is 3.55 × 10^2^ (W/m^2^·μm, 37 °C) [18].

### 2.2. Participants and Study Design

Twenty healthy adults aged 20 years and older, including 10 men and 10 women residing in Busan, Korea, were recruited for the study. The inclusion and exclusion criteria for the participants are provided in Table 1. The participants voluntarily provided written consent after they understood the content form and procedures of the study. Their physical characteristics are presented in Table 2. They used Toloese beds once a day for 3 weeks, excluding weekends, in an environment maintained at 22 ± 2 °C and 45–55% humidity. The Toloese beds were maintained at 37 °C with a hot water boiler. The study participants were instructed to either sleep or rest freely on the bed for one hour, avoid excessive eating but maintain a normal diet. Additionally, they were instructed not to engage in any physical exercise during the 3-week study period. The saliva, blood samples, blood pressure, and BMI of the participants were collected and measured each week before and after they used the Toloese bed. Changes were assessed on the last day of each week. All procedures and experiments were conducted after approval was obtained from the Institutional Review Board (IRB) of Busan Catholic University (IRB: CUPIRB-2021-017).

### 2.3. NO Measurements in Saliva and Serum

Salivary NO was collected in a 50 mL sterile tube and reacted with a NO indicator strip (Knglab, Inc., Seoul, Republic of Korea) for 1 min. Visual color changes were observed in accordance with the manufacturer’s instructions. Serum NO was measured using a nitric oxide colorimetric assay kit (ab65328, Abcam, Cambridge, MA, USA). Serum collected via SST tubes was mixed with nitrate reductase to convert nitrate to nitrite. The absorbance at 540 nm of the azo compound formed by the reaction between Griess reagent and nitrite was measured. NO concentration was expressed as the sum of NO_2_^−^ and NO_3_^−^ (NO_x_), and its detection limit was approximately 1 nmol nitrite/well.

### 2.4. Hematological and Serological Analyses

Blood was collected in EDTA tubes (BD Vacutainer K2 EDTA) and subjected to blood hematological tests. Red blood cell count, white blood cell count, hemoglobin, hematocrit, and platelet count were determined using an automated hematology analyzer, DxH500, Beckman Coulter, Inc., Brea, CA, USA). The blood collected in SST tubes was left to stand for 10 min and then centrifuged at 3500 rpm for 15 min to obtain serum. Serum samples were analyzed using a blood biochemical analyzer (BT 1500; Biotecnica Instruments SpA, Roma, Italy) to measure ALB, GOT, GPT, GGT, ALP, BUN, uric acid, total cholesterol, TG, and LDH.

### 2.5. Blood Pressure, Blood Glucose and BMI Analyses

Blood pressure was measured using a digital automatic blood pressure monitor (MD2680, CAS, Yangju-si, Republic of Korea) on the right arm after a stable seated position was maintained for at least 5 min. Blood glucose levels were determined using the STANDARD™ GlucoNavil^®^ GDH Blood Glucose Monitoring System (01GM30, SD BIOSENSOR, Suwon-si, Republic of Korea) to measure fasting blood glucose levels. BMI was calculated using the height and weight measured with the Smart InBody Body Composition Scale (iGrip, MATIN, Seoul, Republic of Korea), where weight (kg)/height (m^2^). Blood pressure, blood glucose, and BMI were determined before and after the participants used the Toloese bed each week.

### 2.6. Statistical Analysis

Data were statistically analyzed using the SPSS statistical package (ver 27.0) to examine changes in the factors measured in each experiment. Descriptive statistics (mean and standard deviation) were calculated to assess the differences before and after the use of the Toloese bed. A one-way repeated measures ANOVA was conducted to determine significant differences, and data with *p* < 0.05 were considered statistically significant.

## 3. Results

### 3.1. Changes in NO Levels

**Figure 2:**
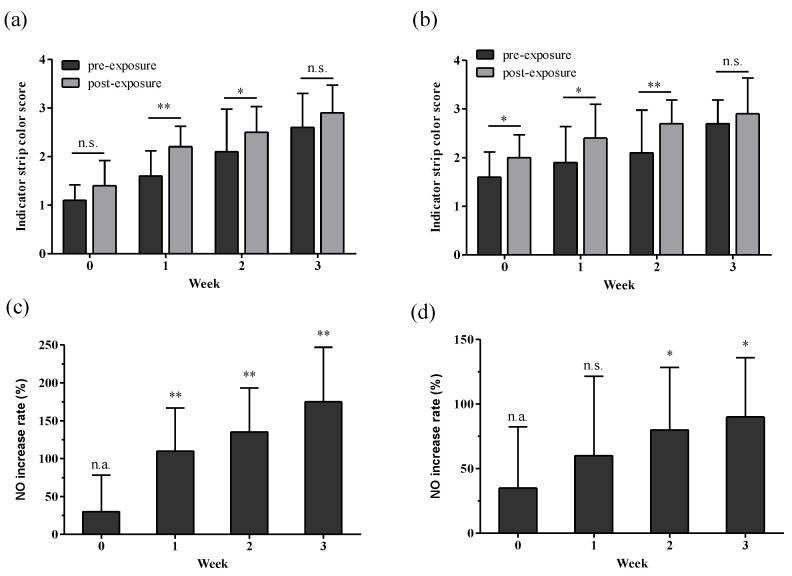
Changes and increased rates of salivary NO before and after using a Toloese bed. For three weeks, 1 h each day, a Toloese Bed at 37 °C was used. (**a**,**b**) represent the changes in salivary NO in males and females, respectively. (**c**,**d**) represent the increase rates of salivary NO after usage compared with pre-usage levels at week 0 in males and females, respectively. Values were expressed as mean ± standard deviation. n.s., non-significant. Paired *t*-tests were used to compare pre- and post-exposure measurements for each week, and one-way ANOVA was used to compare the pre-exposure measurements at week 0 with the post-exposure measurements for each subsequent week. * *p* < 0.05; ** *p* < 0.01.

Changes in salivary NO levels were investigated before and after a Toloese bed was used for 1 h at 37 °C for 3 weeks. NO levels were measured using color changes on strips ranging from 1 to 4 (Figure 2). In the comparison between males and females, all study participants had insufficient NO levels on average. Even after 1 h of use, these levels remained insufficient; however, pre- and post-usage NO levels significantly differed (*p* < 0.05).

In males, the salivary NO levels before and after use in the first and second weeks increased from an average of 1.4 to 1.9 and from 2.4 to 2.7, respectively. However, pre- and post-usage NO levels in the first and second weeks did not significantly differ. In females, NO levels increased from an average of 1.9 to 2.3 in the first week, but they had no significant differences. In the second week, the NO levels before and after use significantly increased from an average of 2.1 to 2.5 (*p* < 0.05). In the third week, the levels in males remained at 2.1 before and after use. NO levels in females increased from 2.3 to 2.6, but they had no significant differences. Salivary NO levels in males and females increased by 118% between the baseline (week 0) and week 3 after using the Toloese bed.

### 3.2. Changes in Serum Nitrite/Nitrate (NOx)

**Figure 3:**
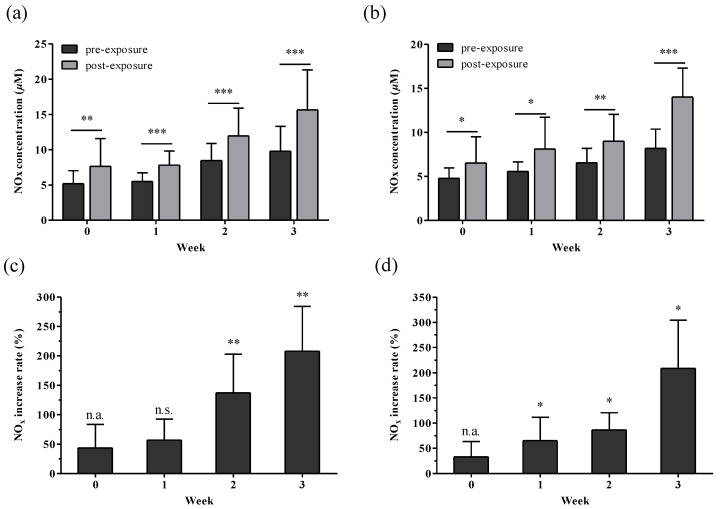
Changes and increased rates of serum NO before and after using a Toloese bed. (**a**,**b**) represent the changes in serum NO in males and females, respectively. (**c**,**d**) represent the increase rates of serum NO after usage compared with pre-usage levels at week 0 in males and females, respectively. Values were expressed as mean ± standard deviation. n.s., non-significant. Paired *t*-tests were used to compare pre- and post-exposure measurements for each week, and one-way ANOVA was used to compare the pre-exposure measurements at week 0 with the post-exposure measurements for each subsequent week. * *p* < 0.05; ** *p* < 0.01; *** *p* < 0.001.

Before and after the Toloese bed was used for 1 h, the serum NO_x_ concentrations at week 0 increased from 7.97 μM to 9.62 μM in males and from 7.50 μM to 8.62 μM in females, but these results had no significant differences (Figure 3).

In the first and second weeks, the NO_x_ concentrations in males before and after use significantly increased from 9.70 μM to 14.50 μM and from 9.21 μM to 13.87 μM, respectively (*p* < 0.01, *p* < 0.05). In the third week, the NO_x_ concentrations significantly increased from 6.97 μM to 10.16 μM after use (*p* < 0.01). In females, the NO_x_ concentrations before and after use in the first and second weeks significantly increased from 7.87 μM to 11.18 μM and from 8.49 μM to 10.98 μM, respectively (*p* < 0.01). In the third week, the NO_x_ concentrations significantly increased from 6.02 μM to 10.38 μM after use (*p* < 0.01). The serum NO_x_ in males and females after using the Toloese bed between weeks 0 and 3 increased by 146% and 172%, respectively.

### 3.3. Changes in Blood Pressure

For each week, changes in systolic and diastolic blood pressure before and after usage of the Toloese bed were measured (Table 3). In males, the systolic blood pressure decreased by 1.65, 2.45, 1.55, and 8.7, and their diastolic blood pressure decreased by 2.5, 5.45, 5.5, and 4.9, respectively. The changes in systolic and diastolic blood pressure did not significantly differ before and after usage. In females, the systolic blood pressure decreased by 5.6, 6.45, 0.9, and 1.4 for each week, and the diastolic blood pressure decreased by 8.6, 1.25, 4.3, and 2.5, respectively. Similar to the changes in males, the changes in systolic and diastolic blood pressure in females were not significantly different before and after usage. Nevertheless, systolic and diastolic blood pressure decreased after usage of the Toloese bed regardless of gender.

### 3.4. Changes in Blood Glucose Levels

Blood glucose significantly decreased in all study participants regardless of gender after they used the Toloese bed (Table 4). In males, these levels decreased by 8.57 ± 5% in week 0; these levels also significantly decreased by 11.58 ± 5%, 6.72 ± 5%, and 9.83 ± 6% in weeks 1 to 3, respectively. Similarly, in females, blood glucose levels pre- and post-usage of the Toloese bed significantly decreased each week.

### 3.5. Changes in Blood Components and Serum Biochemical Factors

The comparison between pre- and post-usage of the Toloese bed revealed that changes in hematological parameters remained within normal ranges for all study participants. No significant changes were observed in pre- and post-usage for each week, indicating that the differences were not statistically sifnificant (Table 5 and Table S1).

Serum biochemical factors for all study participants were within normal ranges, and no significant changes occurred before and after Toloese bed usage, indicating that the differences were not statistically significant (Table 6 and Table S2). However, the comparison between pre-usage and week 3 showed that cholesterol, triglycerides (TG), and low-density lipoprotein (LDL) significantly decreased in males and females (Table 7 and Table S3).

### 3.6. Changes in the Sweat Rate after Toloese Bed Usage

**Figure 4:**
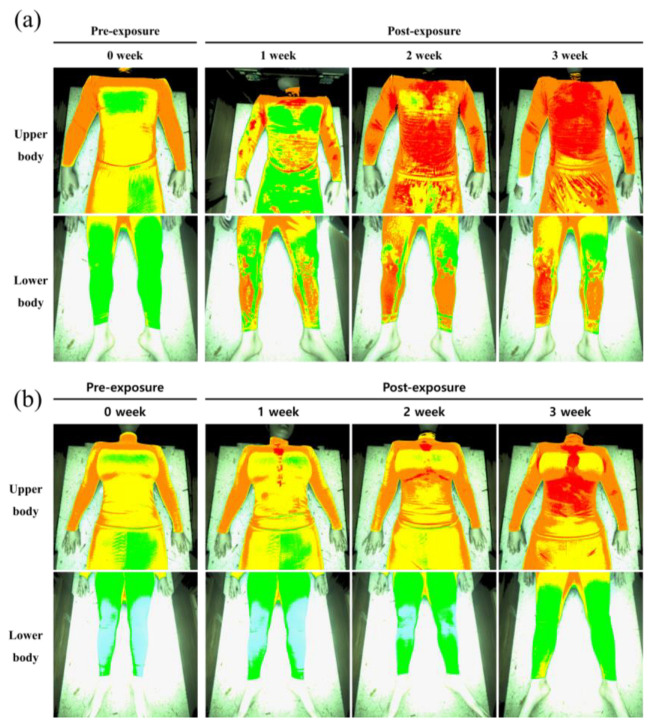
Changes in the sweat rate of the upper and lower body of (**a**) males and (**b**) females after Toloese bed usage in each week. Red indicates a high amount of sweat. Orange indicates a fairly high amount of sweat. Yellow indicates a moderate amount of sweat. Light green indicates a slight amount of sweat. Green indicates a low amount of sweat. Blue indicates almost no sweat.

**Figure 5:**
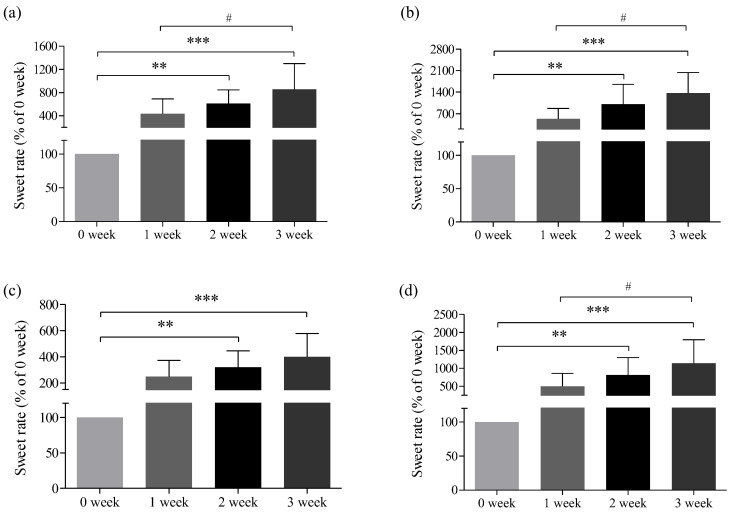
Changes in the sweat rate before and after Toloese bed usage. (**a**,**b**) represent the changes in the exhalation rate of the upper and lower body of males, respectively. (**c**,**d**) depict the changes in the sweat rate of the upper and lower body of females in each week. Values were expressed as mean ± standard deviation. One-way ANOVA was used to compare the pre-exposure measurements at week 0 with the post-exposure measurements for each subsequent week. ** *p* < 0.01; *** *p* < 0.001 for 0 week vs. each week. # *p* < 0.05 for 1 week vs. 3 weeks.

The changes in the exhaled breath before and after Toloese bed usage compared with those in week 0 revealed that the exhalation rates of the upper and lower body gradually increased for all study participants (Figure 4 and Figure 5). In males, the exhalation rates of the upper body increased by 432%, 612%, and 855% in weeks 1, 2, and 3, respectively. The rates observed in weeks 2 and 3 significantly differed from those in week 0, but those in week 1 did not significantly vary (*p* < 0.01, *p* < 0.001). The exhalation rates of the lower body increased by 536%, 1010%, and 1362% in weeks 1, 2, and 3, respectively; significant differences were observed except in week 1 (*p* < 0.01, *p* < 0.001). In females, the exhalation rates of the upper body increased by 250%, 319%, and 401% in weeks 1, 2, and 3, respectively, and the exhalation rates of the lower body increased by 500%, 810%, and 1139% in weeks 1, 2, and 3, respectively. The exhalation rates of the upper and lower body significantly differed in weeks 2 and 3 (*p* < 0.01, *p* < 0.001).

### 3.7. Changes in BMI

Changes in weight and BMI were compared after Toloese bed usage for 1 h. In males, the weight was maintained at 71.5 ± 14.0 kg to 71.7 ± 14.1 kg for 3 weeks, and the BMI was still 24.3 ± 4.7 kg/m^2^. This result indicated that no significant changes occurred after Toloese bed usage. Similarly, the weight and BMI of females did not significantly change between week 0 and week 3 (Table 8).

## 4. Discussion

FIR refers to the wavelength range of 4–1000 μm within the infrared spectrum; the 4–16 μm range of FIR is called growing light rays [19]. It is emitted from all objects with temperatures above absolute zero, including the human body at normal temperatures. It can be absorbed by the human body because of its high water content; thus, FIR can penetrate the dermal layer (4–5 cm) [17,19]. When absorbed, it is converted into thermal energy, primarily affecting the human body through thermogenic actions. It has been used in medical and physical therapies to treat and alleviate various pathologies and diseases [19,20]. In the present study, the Toloese bed was manufactured with an optimal blend of five natural mineral compounds, emitting FIR maximally within a specific wavelength range at temperatures similar to body temperature, ranging from 37 °C to 40 °C. Thus far, FIR therapy has been described in studies conducted in test tubes or based on animal models [21,22]. However, the present study aimed to investigate the correlation between naturally emitted FIR and the increase in NO within the body among healthy adults on a Toloese bed at 37 °C and to analyze changes in blood pressure, hematological, and serum parameters. It involved 10 men and 10 women residing in the Busan area and using a Toloese bed once daily for 5 days over a period of 3 weeks.

NO is a molecule secreted from the inner walls of blood vessels and acts as a signaling agent that dilates the vessels [2]. It plays a crucial role in dilating arteries, maintaining normal blood pressure, and ensuring smooth blood flow to vital organs [23]. The intake of arginine-rich foods reportedly increases the NO concentration in the body [24]. Depending on exercise type, NO levels vary; specifically, serum NO concentrations after aerobic exercise are significantly higher than those after resistance exercise [25]. In the present study, saliva and serum NO significantly increased in week 3 compared with those in week 0 (*p* < 0.001) regardless of gender. This finding suggested that similar to food intake or exercise, Toloese bed usage affected NO generation in the body.

Blood vessels become more elastic and flexible when NO increases in the body; consequently, vascular function is enhanced, and cardiovascular health is promoted [26,27]. In young and healthy individuals, NO can be generated through L-arginine oxidation by NOS [28]. However, with aging, the production of NO derived from NOS decreases, and this decrease has been associated with an increased risk of aging-related hypertension and diabetes [14,15,29]. Despite recent advancements in diagnostic and treatment technologies, the number of patients with hypertension has significantly increased. Most patients with hypertension remain a major clinical concern as the cause often remains unidentified, leading to inadequate or incomplete treatment [14,30].

This study found that systolic and diastolic blood pressure significantly decreased after Toloese bed usage. Additionally, blood glucose levels significantly decreased after Toloese bed usage regardless of gender. Furthermore, all participants in the study exhibited normal hematological and serum test results, and Toloese bed usage did not cause abnormalities in the liver and kidneys. These results suggested that the FIR of the Toloese bed, which increases NO levels in the body, can contribute safely and effectively to blood pressure and blood glucose management.

FIR is utilized for thermal therapy through resonant absorption and vibration resonance, particularly in physical therapy, including various electrotherapy methods such as light therapy, heat and moisture therapy, and high-temperature immersion baths [18,19,31]. FIR thermal therapy provides various physiological effects, including an increase in deep tissue temperature, promotion of blood circulation through vasodilation, and significant reduction in cholesterol and LDL levels [32,33,34,35]. Middle-aged women experienced a significant reduction in total cholesterol after they used a medical-grade far infrared sunbeam capsule at 70–80 °C for 40 min [36].

Similar to previous studies, the present study demonstrated that the total cholesterol and LDL levels significantly decreased regardless of gender after Toloese bed usage at 37 °C for 1 h. Moreover, triglycerides significantly decreased after Toloese bed usage. This result indicated its potential to mitigate factors that could induce conditions such as hyperlipidemia. However, this study targeted healthy individuals; further research targeting patients with diabetes or hyperlipidemia should be performed.

Overall, the Toloese bed contributed to an increase in in-body NO and significantly reduced blood pressure, blood glucose, cholesterol, triglycerides, and LDL levels. Therefore, infrared rays emitted by the Toloese bed might help prevent conditions such as hyperlipidemia, diabetes, and hypertension. However, this study was limited to healthy adults residing in the Busan region, and it was a small-scale observational study with few research subjects. Therefore, follow-up studies targeting patients with cardiovascular diseases or other relevant conditions should be conducted to elucidate the relationship between the increase in in-body NO induced by the Toloese bed’s infrared rays and the changes in hematological and serum parameters.

## 5. Conclusions

This study was conducted to investigate the effects of naturally emitted FIR from a Toloese bed on the generation of endogenous NO in the body. Twenty healthy adult males and females residing in Busan participated in the study, including ten male participants and ten female participants who used the Toloese bed for 1 h daily for 3 weeks. The comparison before and after Toloese bed usage revealed that salivary and serum levels of endogenous NO significantly increased in men and women. Furthermore, systolic and diastolic blood pressure significantly decreased. No hematological or serum biochemical changes were observed, and no adverse effects associated with Toloese bed usage were reported. Additionally, pre-existing levels of blood glucose, cholesterol, triglycerides, and LDL cholesterol significantly decreased after Toloese bed usage. These findings suggested that FIR emitted by Toloese beds might induce the production of endogenous NO. Therefore, Toloese bed usage could be potentially beneficial to the prevention of hypertension, diabetes, and hyperlipidemia.

## Figures and Tables

**Table 1:** Inclusion and exclusion criteria.

Criterion	Details
Inclusion criteria	Adults aged 20 years and older Healthy individuals 10 men and 10 women Individuals able to visit our institution consistently for three weeks
Exclusion criteria	Individuals exhibiting symptoms of anemia Individuals with hypotension Individuals with diabetes Individuals with hyperlipidemia Individuals with other metabolic disorders Individuals on medication affecting lipid or glucose metabolism Pregnant or lactating women Individuals with any chronic illnesses that could affect the study outcomes Individuals who have recently undergone major surgery or medical procedures Individuals with a history of cardiovascular diseases

**Table 2:** General characteristics of the subject.

Variable	Male (*n* = 10)	Female (*n* = 10)
Age (year)	24.4 (±0.9)	21.9 (±2.1)
Height (cm)	171.7 (±4.8)	161.0 (±3.1)
Weight (kg)	71.5 (±14.0)	55.4 (±7.4)
Body mass index	24.3 (±4.7)	21.4 (±2.5)
Fat mass (kg)	15.8 (±9.6)	17.0 (±2.9)

Values are mean ± standard deviation.

**Table 3:** Changes in systolic and diastolic blood pressure before and after Toloese bed usage.

	Week	Sex (*n* = 10)	Change in Blood Pressure (mmHg) *	*T*-Value	*p*-Value
Systolic Blood Pressure	0	Male	4.40 ± 2.37	5.558	0.0004 ***
		Female	8.80 ± 6.42	4.115	0.0026 **
	1	Male	4.30 ± 4.17	3.092	0.0129 *
		Female	6.70 ± 4.47	4.493	0.0015 **
	2	Male	6.40 ± 4.59	4.186	0.0024 **
		Female	6.10 ± 4.66	3.929	0.0035 **
	3	Male	9.90 ± 8.38	3.542	0.0063 **
		Female	7.80 ± 5.56	4.205	0.0023 **
Diastolic Blood Pressure	0	Male	7.30 ± 5.04	4.345	0.0019 **
		Female	8.80 ± 8.01	3.296	0.0093 **
	1	Male	6.00 ± 3.77	4.777	0.001 **
		Female	3.77 ± 2.76	4.146	0.0025 **
	2	Male	10.50 ± 5.68	5.547	0.0004 ***
		Female	6.30 ± 5.12	3.692	0.005 **
	3	Male	8.50 ± 5.85	4.357	0.0018 **
		Female	3.90 ± 2.43	4.821	0.0009 ***

Values are presented as mean ± standard deviation. Paired *t*-tests were used to compare pre- and post-exposure measurements for each week. * *p* < 0.05; ** *p* < 0.01; *** *p* < 0.001.

**Table 4:** Changes in blood glucose before and after Toloese bed usage.

Week	Sex (*n* = 10)	Blood Glucose (mg/dL)		Decrease Rate (%)	*T*-Value	*p*-Value
		Pre-Exposure	Post-Exposure			
0	Male	77.30 ± 8.01	70.85 ± 9.79	8.57 ± 5%	5.067	0.0007 ***
	Female	73.48 ± 6.98	66.57 ± 7.71	9.30 ± 7%	3.793	0.0043 **
1	Male	83.84 ± 8.56	74.02 ± 7.58	11.58 ± 5%	6.168	0.0002 ***
	Female	83.77 ± 4.54	80.12 ± 5.40	4.36 ± 4%	1.137	0.2849 n.s.
2	Male	77.20 ± 8.57	72.22 ± 10.33	6.72 ± 5%	4.318	0.0019 **
	Female	85.71 ± 9.19	79.21 ± 7.93	7.42 ± 5%	4.325	0.0019 **
3	Male	80.79 ± 7.15	72.98 ± 9.33	9.83 ± 6%	4.732	0.0011 **
	Female	85.02 ± 8.62	81.20 ± 7.89	4.40 ± 3%	3.520	0.0065 **

Values are presented as mean ± standard deviation. Paired *t*-tests were used to compare pre- and post-exposure measurements for each week. n.s., not significant; ** *p* < 0.01; *** *p* < 0.001.

**Table 5:** Comparison of hematologic factors before and after Toloese bed usage.

Sex	Parameter	Pre-Exposure	Post-Exposure			
			Week 0	Week 1	Week 2	Week 3
Male (*n* = 10)	RBC (×10^6^/μL)	4.84 (±0.43)	4.79 (±0.4)	4.87 (±0.24)	4.76 (±0.42)	4.87 (±0.28)
	HGB (g/dL)	14.83 (±0.95)	14.65 (±0.83)	14.75 (±0.65)	14.28 (±1.19)	14.55 (±0.87)
	HCT (%)	49.63 (±5.89)	49.31 (±4.81)	45.64 (±2.54)	44.91 (±5.03)	45.46 (±2.28)
	WBC (×10^3^/μL)	4.24 (±1.4)	3.66 (±1.33)	4.33 (±1.08)	4.64 (±1.48)	5.50 (±1.84)
	RLT (×10^3^/μL)	206.18 (±56.72)	200.39 (±55.95)	236.49 (±73.5)	252.41 (±67.15)	232.24 (±53.48)
Female (*n* = 10)	RBC (×10^6^/μL)	4.47 (±0.36)	4.39 (±0.36)	4.30 (±0.37)	4.34 (±0.41)	4.38 (±0.33)
	HGB (g/dL)	12.85 (±1.51)	12.56 (±1.56)	12.24 (±1.08)	12.39 (±1.22)	11.84 (±1.44)
	HCT (%)	42.03 (±2.68)	42.50 (±4.64)	41.80 (±4.19)	42.72 (±4.95)	39.40 (±2.82)
	WBC (×10^3^/μL)	4.79 (±1.8)	4.57 (±1.97)	4.08 (±1.49)	3.93 (±1.52)	5.03 (±1.21)
	RLT (×10^3^/μL)	261.00 (±67.04)	237.57 (±84.92)	268.69 (±97.02)	245.98 (±110.01)	250.06 (±124.36)

Values are presented as mean ± standard deviation.

**Table 6:** Comparison of serum biochemical parameters before and after Toloese bed usage.

Sex	Parameter	Pre-Exposure	Post-Exposure			
			Week 0	Week 1	Week 2	Week 3
Male (*n* = 10)	ALB (g/dL)	4.90 (±0.18)	4.82 (±0.18)	4.78 (±0.31)	4.68 (±0.29)	4.80 (±0.31)
	AST (U/L)	21.37 (±5.54)	20.61 (±5.35)	19.15 (±4.24)	20.13 (±7.20)	22.44 (±12.68)
	ALT (U/L)	18.60 (±16.21)	16.70 (±14.62)	21.50 (±25.43)	20.80 (±18.36)	20.00 (±13.62)
	GGT (U/L)	16.69 (±4.66)	16.31 (±4.49)	25.18 (±29.40)	21.57 (±16.99)	22.21 (±18.17)
	ALP (U/L)	81.35 (±14.22)	76.61 (±11.33)	78.27 (±13.51)	75.03 (±9.54)	80.26 (±12.0)
	Urea (mg/dL)	5.90 (±1.17)	5.99 (±1.16)	5.77 (±0.93)	5.88 (±1.03)	5.31 (±1.15)
	BUN (mg/dL)	13.88 (±3.13)	14.23 (±3.05)	13.20 (±1.87)	13.74 (±2.67)	13.40 (±2.99)
Female (*n* = 10)	ALB (g/dL)	4.86 (±0.35)	4.89 (±0.24)	5.00 (±0.27)	4.72 (±0.4)	4.86 (±0.38)
	AST (U/L)	20.21 (±10.18)	22.04 (±14.44)	16.67 (±0.95)	17.39 (±3.54)	20.23 (±5.42)
	ALT (U/L)	12.00 (±13.07)	12.10 (±13.37)	8.50 (±4.34)	9.20 (±6.35)	12.20 (±7.67)
	GGT (U/L)	15.82 (±7.13)	15.71 (±7.87)	13.71 (±4.03)	13.05 (±3.93)	13.46 (±4.47)
	ALP (U/L)	89.99 (±52.86)	89.07 (±55.67)	89.47 (±54.48)	87.09 (±51.6)	91.12 (±53.36)
	Urea (mg/dL)	5.90 (±1.17)	5.99 (±1.16)	5.77 (±0.93)	5.88 (±1.03)	5.31 (±1.15)
	BUN (mg/dL)	14.80 (±2.4)	14.34 (±2.59)	18.00 (±14.08)	13.44 (±2.31)	12.91 (±3.31)

Values are presented as mean ± standard deviation.

**Table 7:** Changes in cholesterol and lipid test results attributed to Toloese bed usage.

Sex	Parameter	Pre-Exposure	Post-Exposure			
			Week 0	Week 1	Week 2	Week 3
Male (*n* = 10)	CHO (mg/dL)	176.3 ± 25.84	168 ± 27.45	168.1 ± 27.22	163.9 ± 22.48	164.6 ± 22.39
	TG (mg/dL)	82.3 ± 41.33	75 ± 39.12	73.1 ± 29.67	70.3 ± 22.84	78.9 ± 27.04
	HDL-C (mg/dL)	55 ± 7.03	53.1 ± 9.11	54.3 ± 7.01	52 ± 7.46	51.5 ± 9.06
	LDL (mg/dL)	107.8 ± 30.49	99.9 ± 31.25	99.2 ± 26.23	97.8 ± 25.07	97.4 ± 20.61
Female (*n* = 10)	CHO (mg/dL)	179.6 ± 43.7	182.6 ± 40.9	175.6 ± 44.24	170.6 ± 34.62	170.1 ± 33.54
	TG (mg/dL)	66.9 ± 41.59	57.9 ± 27.34	50.7 ± 21.63	57.7 ± 17.69	65.7 ± 21.05
	HDL-C (mg/dL)	66.1 ± 15.4	64.1 ± 13.24	67.8 ± 13.7	61 ± 17.55	61.1 ± 13.71
	LDL (mg/dL)	100.1 ± 26.4	106.9 ± 22.68	97.7 ± 27.85	98.1 ± 22.08	95.9 ± 22.1

Values are presented as mean ± standard deviation.

**Table 8:** Changes in weight and BMI after Toloese bed usage.

Parameter	Male (*n* = 10)				Female (*n* = 10)			
	Week 0	Week 1	Week 2	Week 3	Week 0	Week 1	Week 2	Week 3
BW (kg)	71.5 (±14.0)	71.7 (±14.0)	72.1 (±13.9)	71.7 (±14.1)	55.4 (±7.4)	55.1 (±7.4)	55.2 (±7.3)	55.4 (±6.8)
BMI (kg/m^2^)	24.3 (±4.7)	24.4 (±4.7)	24.4 (±4.6)	24.3 (±4.7)	21.4 (±2.5)	21.3 (±2.5)	21.3 (±2.4)	21.4 (±2.3)

Values are presented as mean ± standard deviation.

## Data Availability

Data are contained within the article.

## Supplementary Materials

The following supporting information can be downloaded at: https://www.mdpi.com/article/10.3390/healthcare12121227/s1, Supplementary Table S1: Comparison of *T*-values and *p*-values for CBC changes before and after using the Toloese bed each week; Supplementary Table S2: Comparison of *T*-values and *p*-values for LFT changes before and after using the Toloese bed each week; Supplementary Table S3: Comparison of *T*-values and *p*-values for cholesterol and lipid changes before and after using the Toloese bed each week.
