# Supplementary material for: Toloese Generates Nitric Oxide through Natural Radiation of Far Infrared Rays, Reducing Serum Glucose, Cholesterol, and Triglycerides
Source: Healthcare (Basel). 2024 Jun 20;12(12):1227. doi: 10.3390/healthcare12121227 (PMC11202990; doi:10.3390/healthcare12121227)
Supplement: Supplementary file 1 [file healthcare-12-01227-s001.zip › healthcare-3037543-supplementary.pdf]

Supplementary Table S1. Comparison of T-values and P-values for CBC changes before and after using the Toloese bed at each week.

Sex	Parameter	Week 0	Week 1	Week 2	Week 3
Male (*n*=10)	RBC	T: 1.147, P: 0.281	T: -0.268, P: 0.795	T: 1.229, P: 0.250	T: -0.222, P: 0.829
	HGB	T: 1.713, P: 0.121	T: 0.223, P: 0.828	T: 3.027, P: 0.014*	T: 0.649, P: 0.532
	HCT	T: 0.564, P: 0.587	T: 2.249, P: 0.051	T: 2.592, P: 0.029*	T: 1.736, P: 0.117
	WBC	T: 2.374, P: 0.042*	T: -0.237, P: 0.818	T: -0.869, P: 0.407	T: -1.918, P: 0.087
	RLT	T: 0.717, P: 0.492	T: -1.319, P: 0.220	T: -1.843, P: 0.098	T: -1.223, P: 0.252
Female (*n*=10)	RBC	T: 1.498, P: 0.168	T: 1.830, P: 0.101	T: 0.962, P: 0.361	T: 0.562, P: 0.588
	HGB	T: 1.886, P: 0.092	T: 2.200, P: 0.055	T: 1.299, P: 0.226	T: 2.007, P: 0.076
	HCT	T: -0.274, P: 0.790	T: 0.127, P: 0.902	T: -0.320, P: 0.756	T: 1.606, P: 0.143
	WBC	T: 0.559, P: 0.590	T: 0.966, P: 0.359	T: 1.119, P: 0.292	T: -0.322, P: 0.755
	RLT	T: 1.351, P: 0.210	T: -0.331, P: 0.748	T: 0.564, P: 0.587	T: 0.282, P: 0.784

T : T-value, P : P-value; * indicates $P < 0.05$.

Supplementary Table S2. Comparison of T-values and P-values for LFT changes before and after using the Toloese bed at each week.

Sex	Parameter	Week 0	Week 1	Week 2	Week 3
Male (*n*=10)	ALB	T: 0.981 P: 0.340	T: n.s. P: n.s.	T: 1.891 P: 0.078	T: 0.869 P: 0.399
	AST	T: 0.296 P: 0.771	T: n.s. P: n.s.	T: 0.409 P: 0.687	T: -0.232 P: 0.820
	ALT	T: 0.261 P: 0.797	T: n.s. P: n.s.	T: -0.269 P: 0.791	T: -0.198 P: 0.845
	GGT	T: 0.174 P: 0.864	T: n.s. P: n.s.	T: -0.832 P: 0.424	T: -0.884 P: 0.397
	ALP	T: 0.782 P: 0.445	T: n.s. P: n.s.	T: 1.107 P: 0.285	T: 0.176 P: 0.863
	Urea	T: -0.160 P: 0.875	T: n.s. P: n.s.	T: 0.029 P: 0.977	T: 1.082 P: 0.294
	BUN	T: -0.239 P: 0.814	T: n.s. P: n.s.	T: 0.102 P: 0.920	T: 0.333 P: 0.743
Female (*n*=10)	ALB	T: -0.177 P: 0.861	T: n.s. P: n.s.	T: 0.795 P: 0.437	T: 0.035 P: 0.973
	AST	T: -0.311 P: 0.760	T: 1.038 P: 0.326	T: 0.785 P: 0.449	T: -0.005 P: 0.996
	ALT	T: -0.016 P: 0.987	T: 0.762 P: 0.462	T: 0.578 P: 0.573	T: -0.040 P: 0.969
	GGT	T: 0.031 P: 0.976	T: 0.773 P: 0.452	T: 1.021 P: 0.325	T: 0.842 P: 0.413
	ALP	T: 0.036 P: 0.972	T: 0.021 P: 0.984	T: 0.118 P: 0.908	T: -0.045 P: 0.964
	Urea	T: 0.301 P: 0.767	T: -0.099 P: 0.922	T: 0.675 P: 0.509	T: 0.737 P: 0.474
	BUN	T: 0.390 P: 0.701	T: -0.672 P: 0.518	T: 1.224 P: 0.237	T: 1.387 P: 0.184

T : T-value; P : P-value; n.s. : not significant.

Supplementary Table S3. Comparison of T-values and P-values for cholesterol and lipid changes before and after using the Toloese bed at each week.

Sex	Parameter	Week 0	Week 1	Week 2	Week 3
Male (*n*=10)	CHO	T: 3.309 P: 0.0091**	T: 2.988 P: 0.0152*	T: 4.321 P: 0.0019**	T: 5.198 P: 0.0006***
	TG	T: 3.816 P: 0.0041**	T: 3.790 P: 0.0043**	T: 4.334 P: 0.0019**	T: 3.846 P: 0.0039**
	HDL-C	T: 2.213 P: 0.054	T: 0.408 P: 0.695	T: 1.441 P: 0.193	T: 2.282 P: 0.057
	LDL	T: 0.356 P: 0.730	T: 2.039 P: 0.081	T: 1.574 P: 0.160	T: 1.259 P: 0.248
Female (*n*=10)	CHO	T: 2.379 P: 0.0413*	T: 2.919 P: 0.0171*	T: 0.975 P: 0.355	T: 3.850 P: 0.0039**
	TG	T: 2.345 P: 0.0437*	T: 4.500 P: 0.0015**	T: 2.753 P: 0.0224*	T: 5.099 P: 0.0006***
	HDL-C	T: 0.296 P: 0.774	T: -0.883 P: 0.4	T: 1.025 P: 0.332	T: 0.63 P: 0.544
	LDL	T: -0.887 P: 0.398	T: 0.320 P: 0.756	T: 0.151 P: 0.884	T: 0.330 P: 0.749

T : T-value; P : P-value; n.s. : not significant.
